# Cell therapy using tolerogenic dendritic cells in transplantation

**DOI:** 10.1186/2047-1440-1-13

**Published:** 2012-09-28

**Authors:** Aurélie Moreau, Emilie Varey, Laurence Bouchet-Delbos, Maria-Cristina Cuturi

**Affiliations:** 1 INSERM, U1064, ITUN, CHU HôtelDieu, 30 Boulevard Jean Monnet, NANTES, France

**Keywords:** Clinical trial, Human, Tolerogenic dendritic cells, Transplantation

## Abstract

Organ transplantation is the main alternative to the loss of vital organ function from various diseases. However, to avoid graft rejection, transplant patients are treated with immunosuppressive drugs that have adverse side effects. A new emerging approach to reduce the administration of immunosuppressive drugs is to co-treat patients with cell therapy using regulatory cells. In our laboratory, as part of a European project, we plan to test the safety of tolerogenic dendritic cell (TolDC) therapy in kidney transplant patients. In this mini-review, we provide a brief summary of the major protocols used to derive human TolDC, and then focus on the granulocyte macrophage-TolDC generated by our own team. Proof of safety of TolDC therapy in the clinic has already been demonstrated in patients with diabetes. However, in transplantation, DC therapy will be associated with the administration of immunosuppressive drugs, and interactions between drugs and DC are possible. Finally, we will discuss the issue of DC origin, as we believe that administration of autologous TolDC is more appropriate, as demonstrated by our experiments in animal models.

## Introduction

Dendritic cells (DC) are potent antigen-presenting cells with dual functions; they can be either immunogenic or tolerogenic in nature. Several protocols of human DC generation have been described and both types of DC have clinical applications. Immunogenic DC are used in patients with cancer to reduce tumor development [1]. Tolerogenic DC (TolDC) therapy appears to be a promising strategy for the treatment of autoimmune diseases and transplantation. In this mini-review, we will focus on human TolDC and their potential clinical application.

### Tolerogenic dendritic cells in humans

In the literature, several protocols have been described for the generation of human TolDC. In these studies, TolDC have been derived from monocytes (MoDC) using the cytokines granulocyte macrophage colony-stimulating factor (GM-CSF) and IL-4. However, as described for tolerogenic bone marrow-derived DC (BMDC) in animal models, different drugs or cytokines could be added to GM-CSF/IL-4 culture to manipulate human DC *in vitro,* to obtain TolDC with specific features [2]. Among those methods, TolDC can be generated with vitaminD3 (VitD3). VitD3-treated DC have the properties of tolerogenic DC; the cells are maturation-resistant, produce IL-10 after stimulation and induce a low proliferation of allogeneic T cells [3-5]. More recently, Raïch-Regué *et al*. showed that VitD3-DC generated from the monocytes of healthy volunteers or patients with relapsing remitting multiple sclerosis have similar properties; a semi-mature phenotype, an anti-inflammatory profile and a low capacity to induce allogeneic T cell proliferation [6]. Furthermore, these cells seem to show potential for clinical application since hyporesponsiveness of myelin-reactive T cells from patients with relapsing remitting multiple sclerosis was observed when these T cells were cultured with autologous TolDC loaded with myelin peptides [6]. In parallel, several studies have investigated the generation of VitD3-TolDC together with dexamethasone (Dex) in order to increase their tolerogenic potential [7]. Prior to the clinical application of these dexamethasone/VitD3 TolDC in rheumatoid arthritis, Harry and colleagues compared the generation of TolDC from healthy volunteers with those from patients with rheumatoid arthritis; a similar phenotype and function was observed between the two groups [8]. In order to favor their migration to the draining lymph nodes and their antigen presentation to T cells, VitD3-DC or Dex/VitD3-DC can be matured *in vitro* with lipopolysaccharide (LPS). Such cells are described as alternatively activated DC [9,10] and induce memory T cell hyporesponsiveness and naive T cell proliferation associated with low IFN-γ and high IL-10 production [9]. Other maturation stimuli such as a cytokine cocktail or monophosphoryl lipid A have also been analyzed [11].

By contrast to Dex-DC and VitD3-DC, rapamycin-treated DC (Rapa-DC) express CD83 and CD86 markers and produce low amounts of IL-10 and high levels of IL-12p40/p70, characteristics of a mature DC phenotype [12]. However, Rapa-DC induce low-level proliferation of allogeneic T cells, similar to Dex-DC and VitD3-DC [13]. Furthermore, Rapa-DC secrete high levels of IL-12 after LPS stimulation, thereby promoting the induction of Treg Foxp3^+^ cells in mice [14]^a^.

Another important molecule used to generate tolerogenic DC is IL-10. Two protocols have been used and lead to the differentiation of different types of TolDC depending on whether IL-10 is present from the initiation of culture or added at the end. In fact, DC generated with IL-10 added at the end of culture have an immature phenotype and display resistance to maturation stimuli [15,16]. These DC induce a state of anergy in CD4^+^ T cells [16] and CD8^+^ T cells [17] in an antigen-specific manner [18]. More recently, DC derived from macaque monocytes in the presence of VitD3 and IL-10 were described as having tolerogenic properties, including resistance to maturation and low-level induction of T cell proliferation [19]. The authors demonstrated the safe intravenous injection of these DC to major histocompatibility complex (MHC)-mismatched recipient macaques treated with antihistamine drug and CTLA4Ig (Cytotoxic T lymphocyte Antigen-4 Ig). A transient increase in donor antigen-specific T cell proliferation was detected in these animals without any increase in anti-donor antibodies [19]. Another protocol to generate TolDC with IL-10 consists of culturing monocytes with IL-10 (in addition to GM-CSF and IL-4) from the initiation of culture. In this case, TolDC (called DC10) express CD83, CD80 and CD86, similar to activated/mature cells, but also Ig-like transcript (ILT)2,ILT3, ILT4 and human leukocyte antigen G, similar to Tol-DC. Furthermore, DC10 secrete high levels of IL-10 and induce hyporesponsiveness in allogeneic T cells [20]. A key characteristic of DC generated with IL-10 is their ability to induce the differentiation of Tr1 regulatory T cells [20,21]^b^. Unfortunately, another property of IL-10-producing DC is a decreased trafficking of these cells to the lymph nodes. The chemokine CCR7 participates in the migration of DC to the lymph nodes, and generating mouse DC with IL-10 down-regulates their expression of CCR7 and impairs their *in vivo* homing to lymph nodes [22]. In a model of mouse cardiac allotransplantation, Garrod *et al*. showed that injection of DC co-expressing IL-10 and CCR7 induced a significant prolongation of graft survival. However, DC expressing either IL-10 only or CCR7 only had no effect [23].

Alternative protocols to generate tolerogenic antigen-presenting cells have been described by other teams, with the resulting cells being referred to as myeloid-derived suppressor cells [24]^c^, mesenchymal stem cells [25,26]^d^ or regulatory macrophages [27]^e^. Clinical trials in transplantation using mesenchymal stem cells and regulatory macrophages have already been performed.

From the studies described in this section, some TolDC were generated using a clinical grade protocol prior to an application in the clinic [6,8,11,13]. In our center, we chose to generate human TolDC using a simple protocol in which monocytes are cultured with low-dose GM-CSF without any additional cytokines or drugs. This protocol is in accordance with our work performed in mice [28] and is compatible with a clinical approach.

### Generation of human granulocyte macrophage tolerogenic dendritic cells

Over the last few years, we have generated and characterized tolerogenic BMDC in rats, mice and non-human primates. In these different models, injection of tolerogenic BMDC leads to a reduced immune response *in vivo* or an induction of tolerance in transplant models [29-31]. Based on this expertise in TolDC generation in animals, we decided to derive TolDC in humans from monocytes in the presence of GM-CSF only. Indeed, the conventional cytokines used to derive dendritic cells from precursors are GM-CSF and IL-4. However, a study performed in mice in 2000 showed that DC generated with a low dose of GM-CSF in the absence of IL-4 have the properties of immature tolerogenic DC. These cells have a high capacity of antigen capture and presentation and induce a low proliferation of allogeneic T cells. Furthermore, they are maturation-resistant and lead to an increase of graft survival after *in vivo* injection [32]. In parallel, more recently, human MoDC generated in the presence of GM-CSF and without IL-4 were described to have tolerogenic properties *in vitro*[33]. Human GM-TolDC are derived from monocytes (0.5 million/mL) cultured with a low dose of GM-CSF (100 U/ml) for 6 days. Different doses of GM-CSF were tested and the best tolerogenic phenotype was obtained in the presence of a low dose. In our protocol, medium and cytokines do not have to be renewed as no difference in the phenotype or function of the cells was observed with or without medium and/or cytokines replacement. On day 6, cells are harvested and characterized on their phenotype and their function.

**Figure 1:**
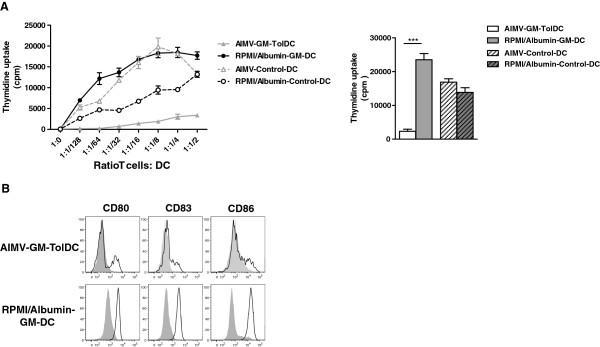
**Influence of culture medium on human dendritic cell differentiation*in vitro.*** Four populations of DC were generated in two different culture media, either RPMI/albumin or AIMV and with two different cytokine conditions, either low-dose GM-CSF (GM-DC) or GM-CSF and IL-4 (Control DC) for 6 days. (**A**) The four DC populations were cultured with allogeneic T cells at different ratios for 6 days. A representative experiment is shown on the left-hand panel. On the right, the results of the ratio of one DC to four Tcells are expressed as the mean T cell proliferation + standard error of the mean for three different donors (*** *P* < 0.001, paired T tests). (**B**) The four populations were un-stimulated (grey solid histogram) or stimulated with 200 ng/mL LPS and 50 ng/mL IFN-γ (black line) for 48 hours. Cell surface expression of CD80, CD83 and CD86 was analyzed after 48 hours of culture by flow cytometry. Data shown are representative of two experiments performed.

To set up this protocol, we tested methods of monocyte selection. The most common method to obtain monocytes is to positively select CD14-positive cells using microbeads. Another possibility is to enrich monocytes from peripheral blood by elutriation. This purification technique is based on the separation of cells according to their size and density [34]. The latter technique, which has been adapted to Good Manufacturing Practice facilities, is much cheaper and isolates less manipulated monocytes. We derived TolDC in AIMV medium (Gibco Life Technologies) and GM-CSF (CellGenix) using these two techniques from the same donor. Analysis of the phenotype, function and maturation resistance of the cells generated from both monocyte isolation protocols gave similar results. Thus, all subsequent experiments were performed with elutriated monocytes. We next tested different clinical-grade culture media. We compared GM-TolDC cultured with GM-CSF alone (100 U/mL) in Roswell Park Memorial Institute (RPMI)/human albumin medium and in AIMV medium for six days. In the literature, CellGroDC or X-VIVO 15 mediums have also been used to derive human TolDC in clinical grade conditions [8,13]. Control non-tolerogenic DC were generated in parallel in the presence of GM-CSF (100 U/mL) and IL-4 (200 U/mL) in both types of media. After 6 days of culture, the DC were all non-adherent in RPMI/albumin medium whereas half of the cells were adherent in AIMV medium. Analysis of the phenotype and function of the cells revealed major differences as the DC generated with RPMI/albumin did not have tolerogenic properties. As shown in Figure 1A, DC cultured with RPMI/albumin induced a strong proliferation of allogeneic T cells, similar to that observed when T cells were cultured with control DC (either in RPMI/albumin or in AIMV media). By contrast, GM-TolDC induce a very low stimulation of allogeneic T cells, a feature that we described previously in rat and macaque tolerogenic BMDC [29,30]. Furthermore, a higher expression of CD80 was detected at day 6 in RPMI/albumin-GM-DC compared with the AIMV-GM-TolDC. This difference in phenotype was much greater after maturation with LPS/IFNγ, as RPMI/albumin-GM-DC highly over-expressed CD80, CD86 and CD83 (Figure 1B).

In summary, GM-TolDC were more than 90% MHC-II^low^ CD80^low^ CD86^low^ CD40^low^ with less than 2% contamination with T cells, B cells or natural killer cells. This immature phenotype of human TolDC is in accordance with the findings of our previous studies in rats, mice and non-human primates [28-30]. Furthermore, as we showed previously in rats [35], human GM-TolDC also expressed the tolerogenic marker Epstein-Barr virus induced gene 3 protein. As regards to their function, GM-TolDC induced a weak stimulation of allogeneic T cells compared with control DC. We also found these cells to be semi-resistant to maturation induced by LPS/IFNγ (as shown in Figure 1). In terms of cytokine release, GM-TolDC produced IL-10 but no IL-12 when stimulated with LPS/IFNγ. Like their phenotype, the *in vitro* function (that is, T cell proliferation induction, maturation-resistance and cytokine production) of human GM-TolDC was similar to that of tolerogenic BMDC described in animal models [28-30]. The similarities between the *in vitro* features of animal TolDC, whose efficacy has been proven *in vivo*, with those obtained in humans, is encouraging for their potential use in the clinic.

### Clinical application of tolerogenic dendritic cells

Whereas clinical trials using immunogenic DC to treat cancer have been developed over the last 15 years [1,36], TolDC therapy is only just emerging in the clinical arena. This was initiated by the pioneer study published in 2001 demonstrating the safety of injecting autologous immature DC into healthy volunteers [37]. Injections of these DC by the subcutaneous route were well-tolerated without signs of toxicity or development of autoimmunity. Inhibition of antigen-specific effector T cell function and induction of antigen-specific CD8 Tregs *in vivo* were detected in DC-treated volunteers [37,38]. More recently, the first phase I clinical trial using TolDC and including 10 patients was reported in type 1 diabetes [39]. Control DC generated in the presence of GM-CSF and IL-4 were injected into three patients while seven patients received immunosuppressive DC generated in the presence of GM-CSF, IL-4 and antisense oligonucleotides targeting CD40, CD80 and C86 transcripts. In this trial, intra-dermal injections of both control and immunosuppressive autologous TolDC were well-tolerated and safe [39]. Furthermore, two clinical trials in rheumatoid arthritis are on-going, one by Thomas’s group in Australia (University of Queensland) and the other one by Hilkens’s and Isaacs’s group in the UK (University of Newcastle). These different studies highlight the emergence of tolerogenic DC therapy as a new approach to treat autoimmune diseases.

As part of a European project, we plan to test the safety of autologous monocyte-derived TolDC in patients who have had a kidney transplant. To avoid graft rejection, transplant patients receive life-long immunosuppressive drugs (IS). That means that in this clinical trial, TolDC will be injected into patients receiving three IS commonly used in transplantation: tacrolimus, mycophenolate mofetil (MMF) and prednisolone. However, as summarized in Table 1, several studies performed in mice and humans have shown that generation of DC in the presence of these IS modifies the DC phenotype and function. In particular, generation of mouse BMDC with tacrolimus reduces the ability of DC to process and/or present antigens [40,41]. All the drug-treated DC have been shown to induce hypoproliferation of allogeneic T cells. Interestingly, a study published in 2005 showed that neither migration nor survival of injected BMDC were affected by tacrolimus-treatment of recipient rats [42]. Furthermore, co-treatment with donor DC and tacrolimus increased the hyporesponsiveness of recipient T cells. In a model of heart allotransplantation, donor DC alone did not delay rejection while tacrolimus or tacrolimus + donor DC induced a prolongation of graft survival [42]. This work was performed using donor-derived mature BMDC. Prior to the clinical trial, we will test the absence of toxicity of our TolDC in IS-treated animals in a mouse skin graft model. In parallel, graft survival will be monitored in animals treated with IS without TolDC therapy. So far, we have noted that injection of MMF induces a prolongation of graft survival and injection of TolDC does not impair this effect. In fact, a slight increase in graft survival was actually detected (Segovia *et al*., manuscript in preparation). Similar experiments using the two other IS or the combination of both IS associated or not with DC therapy are on-going. These studies should identify any potential side effects of DC therapy on the IS treatment used in the clinic.

**Table 1:** **Effects of tacrolimus, mycophenolate mofetil and prednisolone in tolerogenic dendritic cells generated *in vitro ***

**Drugs**	**Models**	**Effects of the drugs on DC and therapeutic effects of the modified DC**	**References**
Tacrolimus	Mouse BMDC +/− Tacrolimus	- Reduction in MHC Class I and Class II-restricted presentation of antigen (role of tacrolimus on Ag processing or presentation)	[40,41,43,44]
		- Reduction in pro-inflammatory cytokine (IL-6 and IL-12) secretion and expression of CD40 and CD86 by BMDC after maturation	
		- Tacrolimus-treated BMDC induce a low proliferation of allogeneic T cells	
		- Low CD69 expression and IFN-γ, IL-2 and IL-4 secretion by T cells stimulated with tacrolimus-treated BMDC	
	Human MoDC +/− Tacrolimus	- Low expression of CXCL10	[45-50]
		- Reduction in pro-inflammatory cytokine (TNFα and IL-12) secretion by BMDC after maturation	
		- Decrease in CD83 and CD86 expression after LPS maturation in the presence of high levels of tacrolimus only	
		- Tacrolimus-treated MoDC induce a low level of allogeneic T cell proliferation, in favor of Th2 cells	
		- Low CD69 expression and IFN-γ, IL-2 and IL-4 secretion by T cells stimulated with tacrolimus-treated MoDC (immature or mature)	
Mycophenolate mofetil	Mouse BMDC +/− MMF	- Decrease in co-stimulatory markers CD80, CD86 and CD40 expression	[51,52]
		- Decrease in IL-12 production	
		- MMF-treated BMDC induce a low proliferation of allogeneic T cells	
		- Decrease in DTH response and increase in allograft survival after injection of MMF-treated-BMDC	
	Human MoDC +/− MMF	- Decrease in co-stimulatory marker expression	[53]
		- Induction of LPS-maturation resistance (in terms of both phenotype and cytokine release)	
		- MMF-treated MoDC induce a low proliferation of allogeneic T cells	
		- Reduction in endocytic capacity in mature MMF-treated MoDC (related to mannose receptor expression)	
Prednisolone	Mouse BMDC +/− prednisolone	- IL-10/methylprednisolone-treated BMDC increase survival of skin allografts	[54]
	Human MoDC +/− prednisolone	- Increase in endocytic capacity	[55-57]
		- Induction of LPS maturation resistance (absence of CD80/CD86/CD83 up-regulation)	
		- Increase in anti-inflammatory cytokines (IL-10 and TGF-β) and decrease in pro-inflammatory cytokines (IL-6, IL-12, IL-23 and TNFα)	
		- Prednisolone-treated MoDC induce a low-level proliferation of allogeneic T cells (which acquire suppressive functions)	

*BMDC*: bone marrow-derived dendritic cells; *CD*: cluster of differentiation; *CXCL10*: chemokine (C-X-C motif) ligand 10; *IFN-γ*: interferon gamma; *IL*: interleukin; *LPS*: lipopolysaccharide; *MHC*: major histocompatibility complex; *MMF*: mycophenolate mofetil; *MoDC*: monocyte-derived dendritic cells; *TNF α*: tumor necrosis factor alpha.

Others parameters related to DC injections should be considered before doing a clinical trial, such as the time of cell product injection, the numbers of DC injected, and also the number of injections as well as the route of administration. Regarding this last parameter, experiments performed in mice have shown that intravenous injection of Dex/LPS-treated BMDC prolongs cardiac transplant survival whereas subcutaneous injection of the same Dex/LPS-treated BMDC does not increase graft survival [58]. Our preliminary experiments in macaques show that intradermal injection of autologous TolDC prime an immune response while intravenous injection does not (unpublished results). A study also performed in monkeys confirmed the fact that intravenous injection of TolDC is well-tolerated [19].

### The potential of autologous tolerogenic dendritic cells in transplantation

The clinical trials described in the previous section of this review either have already been performed or are on-going in autoimmune diseases. In transplantation, another parameter has to be taken into consideration; this is the question of whether TolDC should be derived from the donor or from the recipient. Most of the studies described in rodents have been performed using donor TolDC or recipient TolDC loaded with donor peptides and administrated one week or more before transplantation [2]. In this last part, we discuss the relevance of using autologous TolDC from a safety and efficacy point of view.

Firstly and very importantly, the risk of donor sensitization due to the presence of a slight contaminant cell product or the destruction of the injected cells by non-self recognition cannot be excluded using donor TolDC therapy [59]; we hypothesize that this risk is minimized using autologous TolDC. Furthermore, to be efficient, donor TolDC (or donor pulsed recipient TolDC) need to be sufficiently activated using LPS or other cytokine cocktails in order to migrate to lymphoid organs and present the antigen to T cells [9-11]. To avoid activation-induced maturation, TolDC are also modified using Dex, VitD3 or IL-10 as described in the first section of this review. By contrast, in accordance with our previous work in rodents [30], autologous TolDC do not require activation or pulsing to be efficient, leading to a reduced risk of cell maturation. In accordance with this hypothesis, the first clinical trial using human TolDC performed with MoDC generated with GM-CSF and IL-4 showed no toxicity and no adverse effects in the patients injected with these DC [39], supporting the theory that autologous TolDC do not become immunogenic after injection, correlated with an absence of maturation of the cells.

Secondly, as regards the efficacy of the cells, some studies performed recently in mice by Morelli’s group demonstrated that injected donor DC die quickly after *in vivo* injection. Even if administration of donor TolDC induces tolerance to a transplant, donor TolDC are unable to directly regulate an immune response *in vivo*[60]. In this context, donor DC mediate their suppressive effects on T cells through endogenous conventional DC from the recipient mouse [61]. We believe the mechanisms of action to be different when using autologous TolDC. Indeed, we detected injected recipient rat TolDC in the spleen at least two weeks post-injection [30]. Studies performed in mice and humans have also shown that DC accumulate in the spleen after intravenous injection [62,63]. Furthermore, our experiments showed that donor-derived MHC ClassII^+^ cells from the graft are present in the spleen of the recipient 3 to 5 days after transplantation. Stainings performed in the spleens of these animals suggest that the donor cells interact with the injected TolDC. Moreover, depletion of graft passenger leukocytes from the donor organ before transplantation prevents any effect of the autologous TolDC injection (Segovia *et al*. submitted manuscript). These results validate the hypothesis that injected autologous un-pulsed TolDC are able to migrate to the spleen where they capture and process the donor antigen from graft passenger leukocytes (Segovia *et al*. submitted manuscript) leading to antigen-specific graft acceptance [31].

## Conclusions

As demonstrated by the clinical trial in patients with diabetes [39], the use of autologous tolerogenic DC appears to be a potential safe method that may promote alloantigen-specific Tcell unresponsiveness and transplant survival. Our experiments performed in animals suggest that, to be efficient, injected autologous TolDC have to be in contact with donor antigens and the administration of TolDC at the time of the graft is important. In the clinical trial, patients will be treated with autologous TolDC at the time of the transplant and will be additionally treated with low-dose IS (tacrolimus, prednisolone, MMF). As this will be a phase I trial, we will test the safety and toxicity of TolDC therapy in transplantation. However, we cannot exclude the possibility that IS will have a negative effect on the function of the injected DC, notably by inhibiting antigen presentation as already observed when TolDC were generated with tacrolimus. An alternative could be to change the drugs used or to wait until a decrease in the immunosuppressive treatment is possible before injecting the TolDC.

### Ethical approval

Human leukapheresis samples were collected from healthy donors following institutional-approved protocols (Etablissement Français du Sang, Nantes, France). All animal experiments were performed under specific pathogen-free conditions in accordance with the European Union Guidelines and in compliance with the ethical rules of the INSERM.

## Endnotes

^a^Camilla Macedo; ^b^Giada Amodio; ^c^Natasa Obermajer; ^d^Martin Hoogduijn and Elke Eggenhofer; ^e^James Hutchinson and Paloma Riquelme participated in The One Study Workshop 2012 and described Rapa-DC, DC10, myeloid-derived suppressor cells, mesenchymal stem cells and regulatory macrophages respectively in other mini-reviews.

## Abbreviations

BMDC: Bone-marrow derived dendritic cells; CD: Cluster of differentiation; DC: Dendritic cells; Dex: Dexamethasone; GM-CSF: Granulocyte macrophage colony-stimulating factor; IFN-γ: Interferon gamma; Ig: Immunoglobulin; ILT: Ig-like transcript; IS: Immunosuppressive drugs; LPS: Lipopolysaccharide; MHC: Major histocompatibility complex; MoDC: Monocyte derived DC; MMF: Mycophenolate Mofetil; Rapa: Rapamycin; RPMI: Roswell Park Memorial Institute; TolDC: Tolerogenic DC; VitD3: Vitamin D3.

## Competing interests

The authors declare that they have no competing interests.

## Authors’ contributions

AM participated in the design of the study and drafted the manuscript. EV helped to draft the manuscript. LBD carried out the cell culture and the different assays. MCC conceived the study, participated in its design and coordination, revised and gave the final approval of the manuscript. All the authors read and approved the final manuscript.
